# Methyl 3-(1*H*-indol-3-yl)propano­ate

**DOI:** 10.1107/S1600536811028595

**Published:** 2011-07-23

**Authors:** Rui-Bin Hou, Dong-Feng Li

**Affiliations:** a School of Chemistry and Life Science, Changchun University of Technology, Changchun 130012, People’s Republic of China

## Abstract

The mol­ecule of the title compound, C_12_H_13_NO_2_, adopts an essentially planar conformation (r.m.s. deviation = 0.057 Å). In the crystal, the mol­ecules are linked by inter­molecular N—H⋯O hydrogen bonds, generating chains along [201].

## Related literature

For the biological activity of indole derivatives, see: Zeynep *et al.* (2005 ▶); Seefeld *et al.* (2003 ▶). For details of the synthesis, see: Pedras & Soledade (2006 ▶).
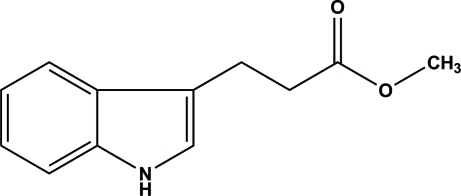


## Experimental

### 

#### Crystal data

C_12_H_13_NO_2_
*M* *_r_* = 203.23
Monoclinic, 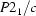

*a* = 6.893 (5) Å
*b* = 9.146 (8) Å
*c* = 18.052 (10) Å
β = 111.27 (3)°
*V* = 1060.5 (13) Å^3^
*Z* = 4
Mo *K*α radiation
μ = 0.09 mm^−1^
*T* = 296 K
0.46 × 0.19 × 0.18 mm

#### Data collection

Rigaku R-AXIS RAPID diffractometer
Absorption correction: multi-scan (*ABSCOR*; Higashi, 1995 ▶) *T*_min_ = 0.961, *T*_max_ = 0.984
10015 measured reflections
2414 independent reflections
1509 reflections with *I* > 2σ(*I*)
*R*_int_ = 0.048

#### Refinement

*R*[*F* ^2^ > 2σ(*F* ^2^)] = 0.052
*wR*(*F* ^2^) = 0.138
*S* = 1.04
2414 reflections
138 parameters
1 restraint
H-atom parameters constrained
Δρ_max_ = 0.16 e Å^−3^
Δρ_min_ = −0.16 e Å^−3^

### 

Data collection: *RAPID-AUTO* (Rigaku Corporation, 1998 ▶); cell refinement: *RAPID-AUTO*; data reduction: *CrystalStructure* (MSC & Rigaku, 2002 ▶); program(s) used to solve structure: *SHELXS97* (Sheldrick, 2008 ▶); program(s) used to refine structure: *SHELXL97* (Sheldrick, 2008 ▶); molecular graphics: *SHELXTL* (Sheldrick, 2008 ▶) and *DIAMOND* (Brandenburg & Berndt, 2001 ▶); software used to prepare material for publication: *SHELXL97*.

## Supplementary Material

Crystal structure: contains datablock(s) I. DOI: 10.1107/S1600536811028595/gk2396sup1.cif

Structure factors: contains datablock(s) I. DOI: 10.1107/S1600536811028595/gk2396Isup2.hkl

Supplementary material file. DOI: 10.1107/S1600536811028595/gk2396Isup3.cml

Additional supplementary materials:  crystallographic information; 3D view; checkCIF report

## Figures and Tables

**Table 1:** Hydrogen-bond geometry (Å, °)

*D*—H⋯*A*	*D*—H	H⋯*A*	*D*⋯*A*	*D*—H⋯*A*
N1—H1⋯O1^i^	0.95	2.08	2.972 (3)	157

Symmetry code: (i) 
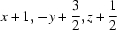
.

**Fig. 1.**
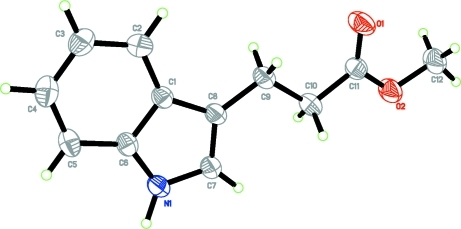
Molecular structure of the title compound with displacement ellipsoids drawn at the 30% probalility level.

**Fig. 2.**
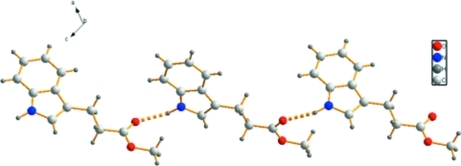
Hydrogen-bonded chain in the title compound. Dashed lines indicate hydrogen bonds.

## supplementary crystallographic information

### Comment

Indole derivatives constitute an important class of therapeutic agents in medicinal chemistry including anticancer, antioxidant, antirheumatoidal and anti-HIV (Zeynep *et al.*, 2005; Seefeld *et al.*, 2003). We have recently synthesized some indole derivatives as histone deacetylase (HDAC) inhibitors with the precursor. In this paper, we report the crystal structure of the title compound.

The molecular structure of tiltle compound, C_12_H_13_O_2_N, as shown in Fig. 1, all bond lengths and angles are in the normal ranges. All non-hydrogen atoms except for O1 are nearly coplanar. In the crystal, the intermolecular N—H···O hydrogen bonds link the molecules into chains along the [201] direction.

### Experimental

The title compound was prepared according to the literature (Pedras & Soledade, 2006). Single crystals suitable for X-ray diffraction were prepared by slow evaporation method from a solution in dichloromethane/ petroleum (60–90 °C) at room temperature.

### Refinement

Carbon-bound H-atoms were placed in calculated positions (C—H 0.93 and 0.97 Å) and were included in the refinement in the riding model with *U*_iso_(H) = 1.5 or 1.2 *U*_eq_(C). The N-bound H atom was located from a difference map and refined with the distance restraint N—H = 0.90 Å and *U*_iso_(H) = 1.5 *U*_eq_(N).

### Crystal data

**Table:**

C_12_H_13_NO_2_	*F*(000) = 432
*M**_r_* = 203.23	*D*_x_ = 1.273 Mg m^−^^3^
Monoclinic, *P*2_1_/*c*	Mo *K*α radiation, λ = 0.71073 Å
Hall symbol: -P 2ybc	Cell parameters from 5849 reflections
*a* = 6.893 (5) Å	θ = 3.2–27.4°
*b* = 9.146 (8) Å	µ = 0.09 mm^−^^1^
*c* = 18.052 (10) Å	*T* = 296 K
β = 111.27 (3)°	Block, colorless
*V* = 1060.5 (13) Å^3^	0.46 × 0.19 × 0.18 mm
*Z* = 4	

### Data collection

**Table:**

Rigaku R-AXIS RAPID diffractometer	2414 independent reflections
Radiation source: fine-focus sealed tube	1509 reflections with *I* > 2σ(*I*)
graphite	*R*_int_ = 0.048
ω scans	θ_max_ = 27.5°, θ_min_ = 3.2°
Absorption correction: multi-scan (*ABSCOR*; Higashi, 1995)	*h* = −8→8
*T*_min_ = 0.961, *T*_max_ = 0.984	*k* = −11→11
10015 measured reflections	*l* = −23→23

### Refinement

**Table:**

Refinement on *F*^2^	Secondary atom site location: difference Fourier map
Least-squares matrix: full	Hydrogen site location: inferred from neighbouring sites
*R*[*F*^2^ > 2σ(*F*^2^)] = 0.052	H-atom parameters constrained
*wR*(*F*^2^) = 0.138	*w* = 1/[σ^2^(*F*_o_^2^) + (0.0614*P*)^2^ + 0.1289*P*] where *P* = (*F*_o_^2^ + 2*F*_c_^2^)/3
*S* = 1.04	(Δ/σ)_max_ < 0.001
2414 reflections	Δρ_max_ = 0.16 e Å^−^^3^
138 parameters	Δρ_min_ = −0.16 e Å^−^^3^
1 restraint	Extinction correction: *SHELXL*, Fc^*^=kFc[1+0.001xFc^2^λ^3^/sin(2θ)]^-1/4^
Primary atom site location: structure-invariant direct methods	Extinction coefficient: 0.036 (5)

### Special details

**Table:**

Experimental. (See detailed section in the paper)
Geometry. All e.s.d.'s (except the e.s.d. in the dihedral angle between two l.s. planes) are estimated using the full covariance matrix. The cell e.s.d.'s are taken into account individually in the estimation of e.s.d.'s in distances, angles and torsion angles; correlations between e.s.d.'s in cell parameters are only used when they are defined by crystal symmetry. An approximate (isotropic) treatment of cell e.s.d.'s is used for estimating e.s.d.'s involving l.s. planes.
Refinement. Refinement of *F*^2^ against ALL reflections. The weighted *R*-factor *wR* and goodness of fit *S* are based on *F*^2^, conventional *R*-factors *R* are based on *F*, with *F* set to zero for negative *F*^2^. The threshold expression of *F*^2^ > σ(*F*^2^) is used only for calculating *R*-factors(gt) *etc*. and is not relevant to the choice of reflections for refinement. *R*-factors based on *F*^2^ are statistically about twice as large as those based on *F*, and *R*- factors based on ALL data will be even larger.

### Fractional atomic coordinates and isotropic or equivalent isotropic displacement parameters (Å^2^)

**Table:**

	*x*	*y*	*z*	*U*_iso_*/*U*_eq_	
O1	−0.1892 (2)	0.69967 (17)	0.43997 (8)	0.0762 (5)	
O2	−0.3213 (2)	0.88674 (17)	0.48330 (8)	0.0760 (5)	
N1	0.5979 (2)	0.80147 (17)	0.76433 (8)	0.0566 (4)	
H1	0.6448	0.8259	0.8192	0.085*	
C1	0.5726 (3)	0.65764 (19)	0.66104 (9)	0.0469 (4)	
C2	0.6478 (3)	0.5573 (2)	0.61952 (11)	0.0585 (5)	
H2	0.5628	0.5253	0.5693	0.070*	
C3	0.8477 (3)	0.5065 (2)	0.65345 (13)	0.0668 (6)	
H3	0.8984	0.4403	0.6258	0.080*	
C4	0.9761 (3)	0.5526 (2)	0.72877 (13)	0.0671 (6)	
H4	1.1109	0.5161	0.7507	0.080*	
C5	0.9079 (3)	0.6506 (2)	0.77134 (11)	0.0598 (5)	
H5	0.9941	0.6810	0.8217	0.072*	
C6	0.7059 (3)	0.70276 (19)	0.73679 (10)	0.0481 (4)	
C7	0.4000 (3)	0.8150 (2)	0.70877 (10)	0.0546 (5)	
H7	0.2961	0.8736	0.7145	0.066*	
C8	0.3779 (3)	0.7306 (2)	0.64415 (9)	0.0491 (5)	
C9	0.1912 (3)	0.7142 (2)	0.56905 (10)	0.0606 (5)	
H9A	0.1467	0.6129	0.5639	0.073*	
H9B	0.2316	0.7372	0.5242	0.073*	
C10	0.0095 (3)	0.8096 (2)	0.56507 (10)	0.0558 (5)	
H10A	−0.0319	0.7868	0.6097	0.067*	
H10B	0.0529	0.9111	0.5699	0.067*	
C11	−0.1734 (3)	0.7903 (2)	0.48976 (10)	0.0532 (5)	
C12	−0.5084 (3)	0.8803 (3)	0.41298 (12)	0.0820 (7)	
H12A	−0.4753	0.9036	0.3671	0.123*	
H12B	−0.6080	0.9494	0.4178	0.123*	
H12C	−0.5661	0.7836	0.4074	0.123*	

### Atomic displacement parameters (Å^2^)

**Table:**

	*U*^11^	*U*^22^	*U*^33^	*U*^12^	*U*^13^	*U*^23^
O1	0.0724 (10)	0.0813 (11)	0.0522 (7)	0.0196 (8)	−0.0047 (7)	−0.0124 (7)
O2	0.0590 (9)	0.0844 (11)	0.0673 (8)	0.0192 (8)	0.0020 (7)	−0.0161 (7)
N1	0.0537 (9)	0.0640 (10)	0.0445 (8)	−0.0005 (8)	0.0087 (7)	−0.0074 (7)
C1	0.0477 (10)	0.0480 (10)	0.0424 (8)	−0.0038 (8)	0.0132 (8)	0.0026 (7)
C2	0.0677 (13)	0.0552 (12)	0.0516 (10)	−0.0013 (10)	0.0204 (9)	−0.0030 (8)
C3	0.0675 (14)	0.0577 (13)	0.0807 (14)	0.0070 (10)	0.0338 (11)	−0.0018 (10)
C4	0.0520 (12)	0.0571 (13)	0.0878 (14)	0.0035 (10)	0.0202 (11)	0.0053 (11)
C5	0.0475 (11)	0.0566 (12)	0.0635 (11)	−0.0053 (9)	0.0060 (9)	0.0001 (9)
C6	0.0458 (10)	0.0459 (10)	0.0486 (9)	−0.0054 (8)	0.0124 (8)	0.0030 (8)
C7	0.0476 (11)	0.0620 (12)	0.0485 (9)	0.0046 (9)	0.0107 (8)	−0.0029 (8)
C8	0.0487 (10)	0.0542 (11)	0.0401 (9)	−0.0019 (8)	0.0109 (8)	−0.0001 (7)
C9	0.0529 (11)	0.0744 (14)	0.0440 (9)	0.0054 (10)	0.0050 (8)	−0.0041 (9)
C10	0.0574 (12)	0.0549 (11)	0.0464 (9)	−0.0020 (9)	0.0084 (8)	0.0004 (8)
C11	0.0542 (11)	0.0548 (11)	0.0460 (9)	0.0027 (9)	0.0126 (8)	0.0045 (8)
C12	0.0555 (13)	0.0987 (19)	0.0722 (13)	0.0191 (12)	−0.0002 (11)	−0.0103 (12)

### Geometric parameters (Å, °)

**Table:**

O1—C11	1.198 (2)	C5—C6	1.388 (3)
O2—C11	1.321 (2)	C5—H5	0.9300
O2—C12	1.446 (2)	C7—C8	1.361 (3)
N1—C6	1.373 (2)	C7—H7	0.9300
N1—C7	1.375 (2)	C8—C9	1.500 (2)
N1—H1	0.9498	C9—C10	1.507 (3)
C1—C2	1.398 (3)	C9—H9A	0.9700
C1—C6	1.404 (2)	C9—H9B	0.9700
C1—C8	1.429 (3)	C10—C11	1.491 (2)
C2—C3	1.371 (3)	C10—H10A	0.9700
C2—H2	0.9300	C10—H10B	0.9700
C3—C4	1.392 (3)	C12—H12A	0.9600
C3—H3	0.9300	C12—H12B	0.9600
C4—C5	1.370 (3)	C12—H12C	0.9600
C4—H4	0.9300		
			
C11—O2—C12	117.67 (16)	C7—C8—C1	106.12 (15)
C6—N1—C7	108.66 (15)	C7—C8—C9	128.62 (17)
C6—N1—H1	120.5	C1—C8—C9	125.26 (16)
C7—N1—H1	127.7	C8—C9—C10	114.27 (16)
C2—C1—C6	118.42 (17)	C8—C9—H9A	108.7
C2—C1—C8	133.97 (16)	C10—C9—H9A	108.7
C6—C1—C8	107.61 (16)	C8—C9—H9B	108.7
C3—C2—C1	119.42 (18)	C10—C9—H9B	108.7
C3—C2—H2	120.3	H9A—C9—H9B	107.6
C1—C2—H2	120.3	C11—C10—C9	112.84 (16)
C2—C3—C4	120.9 (2)	C11—C10—H10A	109.0
C2—C3—H3	119.5	C9—C10—H10A	109.0
C4—C3—H3	119.5	C11—C10—H10B	109.0
C5—C4—C3	121.4 (2)	C9—C10—H10B	109.0
C5—C4—H4	119.3	H10A—C10—H10B	107.8
C3—C4—H4	119.3	O1—C11—O2	122.57 (17)
C4—C5—C6	117.73 (18)	O1—C11—C10	125.61 (18)
C4—C5—H5	121.1	O2—C11—C10	111.82 (16)
C6—C5—H5	121.1	O2—C12—H12A	109.5
N1—C6—C5	130.60 (17)	O2—C12—H12B	109.5
N1—C6—C1	107.26 (15)	H12A—C12—H12B	109.5
C5—C6—C1	122.14 (17)	O2—C12—H12C	109.5
C8—C7—N1	110.32 (16)	H12A—C12—H12C	109.5
C8—C7—H7	124.8	H12B—C12—H12C	109.5
N1—C7—H7	124.8		
			
C6—C1—C2—C3	−0.2 (3)	N1—C7—C8—C1	1.2 (2)
C8—C1—C2—C3	−179.77 (19)	N1—C7—C8—C9	−178.59 (18)
C1—C2—C3—C4	0.5 (3)	C2—C1—C8—C7	179.6 (2)
C2—C3—C4—C5	−0.4 (3)	C6—C1—C8—C7	−0.1 (2)
C3—C4—C5—C6	−0.1 (3)	C2—C1—C8—C9	−0.6 (3)
C7—N1—C6—C5	−178.76 (18)	C6—C1—C8—C9	179.73 (17)
C7—N1—C6—C1	1.8 (2)	C7—C8—C9—C10	2.6 (3)
C4—C5—C6—N1	−179.00 (18)	C1—C8—C9—C10	−177.13 (17)
C4—C5—C6—C1	0.4 (3)	C8—C9—C10—C11	−179.92 (16)
C2—C1—C6—N1	179.23 (15)	C12—O2—C11—O1	0.1 (3)
C8—C1—C6—N1	−1.06 (19)	C12—O2—C11—C10	−179.83 (17)
C2—C1—C6—C5	−0.3 (3)	C9—C10—C11—O1	7.5 (3)
C8—C1—C6—C5	179.44 (16)	C9—C10—C11—O2	−172.55 (16)
C6—N1—C7—C8	−1.9 (2)		

### Hydrogen-bond geometry (Å, °)

**Table:**

*D*—H···*A*	*D*—H	H···*A*	*D*···*A*	*D*—H···*A*
N1—H1···O1^i^	0.95	2.08	2.972 (3)	157

Symmetry codes: (i) *x*+1, −*y*+3/2, *z*+1/2.
